# Effect of Preparation Method on the Catalytic Performance of HZSM-5 Zeolite Catalysts in the MTH Reaction

**DOI:** 10.3390/ma15062206

**Published:** 2022-03-17

**Authors:** Junhua Gao, Hao Zhou, Fucan Zhang, Keming Ji, Ping Liu, Zenghou Liu, Kan Zhang

**Affiliations:** 1 State Key Laboratory of Coal Conversion, Institute of Coal Chemistry, Chinese Academy of Sciences, Taiyuan 030001, China; gaojunhua@sxicc.ac.cn (J.G.); zhouhao@sxicc.ac.cn (H.Z.); 17865422752@163.com (F.Z.); zhliu@sxicc.ac.cn (Z.L.); 2 State Key Laboratory of Heavy Oil Processing, China University of Petroleum, Beijing 102249, China

**Keywords:** ZSM-5, binder, pseudoboehmite, acid, hydrocarbon, coke

## Abstract

A kind of nano-ZSM-5 zeolite crystal was synthesized by the hydrothermal method, and HZSM-5 zeolite powder was obtained via acid exchange. By using pseudoboehmite as a binder, a series of HZSM-5 zeolite catalysts for methanol-to-hydrocarbons (MTH) were prepared through adjusting the binder content between 20 and 50% in addition to the molding method of wet extrusion and mechanical mixing. XRD, ^27^Al NMR, SEM-EDS, ICP, low-temperature N_2_ adsorption and desorption, NH_3_-TPD, Py-FTIR, FT-IR, TG and elemental analyses were used to characterize the properties of fresh catalysts and coke-deposited catalysts. Then, MTH catalytic performance was evaluated in a continuous-flow fixed-bed reactor. The characterization and evaluation results showed that the addition of dilute nitric acid during the molding process increased the amount of moderate-strength acid and formed a hierarchical pore distribution, which helped to reduce the reaction ability of cracking, aromatization and hydrogen transfer, improve the diffusion properties of the catalyst and slow down the coke deposition rate. The catalyst with a binder content of 30% made by wet extrusion with dilute nitric acid had the best performance, whose activity stability of MTH increased by 96 h, higher than other catalysts, and the coke deposition rate was slower, which was due to the most suitable distribution of acid strength and B/L ratio as well as the most obvious hierarchical pore structure.

## 1. Introduction

The conversion of methanol-to-hydrocarbons (MTH), including gasoline, aromatic or olefin, on acidic zeolites is a very important way for fossil fuels and biomass to produce bulk chemicals from syngas; it has become one of the hot topics in the field of catalysis in the past twenty years [1,2]. The present situation of energy in China is that it is rich in coal resources and poor in the resources of oil and natural gas. The technology of methanol production from syngas has been matured, which could provide a good raw material foundation for MTH. ZSM-5 is the most commonly used zeolite catalyst in the MTH process, with a three-dimensional crosspore structure and a high Si/Al ratio; as a result, it has unique shape selectivity, good hydrothermal stability and strong resistance to coke deposition ability [3,4]. ZSM-5 zeolites are usually synthesized by the hydrothermal method in an alkaline sol–gel system, and the product of ZSM-5 zeolites is an alkaline crystal powder containing template and alkali metal cations. The template needs to be removed by drying and calcining, and then the zeolite powder is exchanged with an acid solution or an ammonium salt solution to form hydrogen-type ZSM-5 (HZSM-5) raw powder. In industrial applications HZSM-5 raw powder is molded with a certain amount of binder by wet extrusion, dried and calcined to achieve the mechanical strength required for industrial applications and then the finished MTH catalyst is prepared. The molding of HZSM-5 zeolite raw powder and binders is an important part in the industrial preparation process of an MTH catalyst. This process can not only enhance the strength of the catalyst but also affect the acid active centers and textural properties of the catalyst by adding a binder, thus affecting the reaction performance of the catalyst [5,6,7]. In addition, coke deposition during the MTH reaction is the main reason for the one-way deactivation of the catalyst [8,9,10]. The preparation methods affect the acidity and pore distribution of the catalyst, thus affecting the deactivation behavior of coke deposition. Therefore, it is also of great significance to study the effect of the preparation method on the catalytic performance and coke behaviors of HZSM-5 zeolites for the development of an MTH catalyst in industrial applications.

The typically used binders include silica, alumina, kaolin and so on, which play different roles in the molding process. Pseudoboehmite is a kind of alumina binder used often and commonly in the extrusion molding process of a ZSM-5 zeolite catalyst [11,12,13,14]. In order to investigate the influence of the binder content and molding method on the MTH catalytic performance of ZSM-5 zeolite catalysts, pseudoboehmite was used as the binder to prepare different kinds of catalysts (wet extrusion molding with dilute nitric acid, mechanical mixing pressing molding and wet extrusion molding with water). In our study the effects of the molding method and binder content on the catalytic performance of MTH were discussed, and a catalyst with industrial application value was prepared. The content and C/H ratio of coke deposition on the catalyst were compared, and the influence of the molding method on coke behavior was analyzed.

## 2. Materials and Methods

### 2.1. Catalyst Preparation

ZSM-5 zeolites were synthesized by the hydrothermal method, using sodium silicate (modulus 3.3, Qingdao Dongyue sodium silicate Co., Ltd., Qingdao, China) as a silicon source, aluminum sulfate octadecahydrate (Al_2_(SO_4_)_3_·18H_2_O, analytical purity, Tianjin Beichen Fangzheng Reagent Factory, Tianjin, China) as an aluminum source, tetrapropylammonium bromide as a template (TPABr, chemical purity, Zhejiang Kente Chemical Co., Ltd., Xianju, China) and concentrated sulfuric acid (H_2_SO_4_, chemical purity, Sinopharm Chemical Reagent Co., Ltd., Beijing, China) as a pH regulator, to prepare a certain proportion of gel. The obtained gel was put into a 2 L stainless steel autoclave, and the stirring speed was 400 rev/min. ZSM-5 zeolite crystals were obtained through aging at low temperature and crystallization at high temperature, controlling the synthetic temperature and crystallization time. The obtained crystals were washed to neutral with deionized water, dried at 393 K for 12 h and calcinated at 813 K for 4 h to remove the organic template. According to 50 mL of a 0.5 mol/L ammonium nitrate (NH_4_NO_3_, chemical purity, Sinopharm Chemical Reagent Co., Ltd., Beijing, China) solution of each gram of NaZSM-5 zeolites, the hydrogen-type ZSM-5 zeolite (HZSM-5) raw powder was obtained by exchanging at 353 K for three times. The HZSM-5 raw powder was pressed into tablets and broken into a 20–40 mesh for standby, which was recorded as CZ.

Weigh 8 g, 7 g, 6 g and 5 g of HZSM-5 zeolite powder, then weigh 2 g, 3 g, 4 g and 5 g of pseudoboehmite (Zibo Baida Chemical Co., Ltd., Zibo, China), respectively. Mix them evenly, add dilute nitric acid solution, knead evenly and extrude them. The formed HZSM-5 catalysts with pseudoboehmite contents of 20%, 30%, 40% and 50% were prepared, which were recorded as CN-20, CN-30, CN-40 and CN-50 successively. Weigh 7 g of HZSM-5 zeolite raw powder and 3 g of pseudoboehmite, mix them mechanically, grind them evenly in an agate mortar and press them into tablets, recorded as CP-30. Weigh 7 g of HZSM-5 zeolite raw powder and 3 g of pseudoboehmite, mix them evenly, add deionized water, knead them evenly and extrude them into shape, recorded as CW-30. All the formed catalysts were dried at 393 K for 12 h, calcinated at 813 K for 4 h and broken into a 20–40 mesh for subsequent catalyst evaluation.

### 2.2. Catalyst Characterization

X-ray diffraction (XRD) phase analysis was carried out on a D8 ADVANCE X-ray powder diffractometer (Bruker, Germany). Cu target, Kα ray (λ = 0.15 nm), tube voltage of 40 kV, tube current of 30 mA, 5–50° scanning and step size of 0.02°.

Fourier transform infrared (FT-IR) spectra characterization was carried out on a Bruker Vector 22 infrared spectrometer (Bruker, Germany). The samples and potassium bromide powder were mixed and ground as the mass ratio of 1:(200–400), and then the spectra were scanning at room temperature after forming by pressing.

An ^27^Al NMR test was carried out on an AVANCE IIITM 600 MHz superconducting NMR spectrometer (Bruker, Switzerland). ^27^Al NMR spectra were obtained at 10.0 kHz using a 1 s delay, for a total of 10000 pulses.

The catalysts’ morphology was determined by a JSM-7001F thermal field emission scanning electron microscope (SEM) with a voltage of 10 kV (JEOL, Akishima, Tokyo, Japan). Elemental distributions were acquired with an X-ray energy-dispersive spectrometer (EDS, X-Flash 5010) detector at 15 kV.

Transmission electron microscopy (TEM) images were recorded on a Tecnai G2 F20 S-Twin microscope operated at 200 kV (FEI, Hillsboro, America).

The composition of HZSM-5 zeolites were determined by a Thermo iCAP 6300 inductively coupled plasma (ICP) atomic emission spectrometer (Thermo Fisher, Waltham, America).

Low-temperature N_2_ adsorption and desorption isotherms at 77 K were recorded using a Micromeritics ASAP 2010 instrument (Micromeritics, America). Before the measurements the samples were heated to 570 K in a vacuum for at least 12 h. The specific surface area, mesopore size pore distribution and micropore volume were calculated by the Brunauer–Emmett–Teller (BET) method, the Barret–Joyner–Halenda (BJH) method and the *t*-plot method by Harkins and Jura (DeBoer) thickness equation, with a thickness range of 3.5 to 5 Å, respectively.

Ammonia temperature-programmed desorption (NH_3_-TPD) characterization was carried out on a micro automatic multipurpose adsorption instrument, TP-5080 (Tianjin Xianquan, China). The catalyst was first purged with N_2_ at 773 K for 60 min, with a N_2_ flow rate of 30 mL/min, and then reduced to 373 K for the adsorption of NH_3_. After the baseline was stable the ammonia desorption experiment was conducted at 10 K/min to 973 K.

Pyridine Fourier transform infrared (Py-FTIR) was characterized on a Bruker Vector 22 infrared spectrometer (Bruker, Karlsruhe, Germany). The catalyst was first treated at 673 K and 0.05 Pa for 30 min to remove the adsorbed impurities. Pyridine was adsorbed at room temperature. After desorption at 573 K and 0.05 Pa, the characteristic peaks of Brönsted (B) acid and Lewis (L) acid were integrated. The distribution of B acid and L acid was represented by the ratio of the peak area of the two kinds of acids.

Diffuse reflectance Fourier transform infrared (FT-IR) spectra were measured on a Bruker Tensor 27 FT-IR spectrometer (Bruker, Germany). The catalyst samples were first calcinated at 813 K for 4 h in the muffle furnace. Prior to the measurement the sample was heated at 723 K for 2 h with a N_2_ flow of 15 mL/min in situ cell. The IR spectra were then recorded at room temperature.

C and H elements were analyzed by a Vario EL CUBE element analyzer (Elementar, Frankfurt, Germany).

The thermogravimetry (TG) analysis of deposited coke catalysts was carried out on a Setsys Evolution thermogravimetric analyzer (Setaram, Lyon, France). O_2_ atmosphere, from 303 K to 1173 K, with a heating rate of 10 K/min and a gas flow rate of 20 mL/min.

### 2.3. Catalyst Evaluation

The catalyst was evaluated in a continuous-flow fixed-bed reactor with stainless steel tubes of 100 cm in length and 1 cm in inner diameter. Three grams of catalyst was loaded into the constant temperature section of the reactor, and quartz sand was filled at both ends of the bed. MTH reaction performance was evaluated at atmospheric pressure, 653 K and a methanol weight space time velocity (WHSV) of 4 h^−^^1^. The products were separated by cold trap gas–liquid separation. The gas- and water-phase products were analyzed by an SP-2000 gas chromatograph of a Beijing Beifen Ruili analytical instrument (Group) Co., Ltd. (Beijing, China). The gas-phase products were analyzed by a TDX-102 packed column, a thermal conductivity detector (TCD), an aluminum oxide (Al_2_O_3_) capillary column and a hydrogen flame (FID) detector. A Porapak-Q packed column and TCD detector were used for the analysis of aqueous products. Oil-phase products in the liquid-phase products were analyzed by an SP-3420 gas chromatograph of a Beijing Beifen Ruili analytical instrument (Group) Co., Ltd. The chromatographic columns for oil-phase products were an HP INNOWAX capillary column and an FID detector. The methanol conversion, product distribution and hydrogen transfer index (HTI) are defined as follows:

Methanol conversion *x* = (mass of feed methanol − mass of methanol in aqueous phase)/mass of feed methanol × 100% (1)

Product distribution *s* = mole number of carbon contained in one product/total productcarbon mole number × 100% (2)

Aromatics content in oil phase *c* = mass of some product in C_5_^+^/mass of liquid phase product × 100% (3)

Hydrogen transfer index = V_(propane + butane)_/V_(propylene + butene)_ × 100% (4)

## 3. Results

### 3.1. Catalyst Crystal Structure, ^27^Al NMR and Morphology

**Figure 1:**
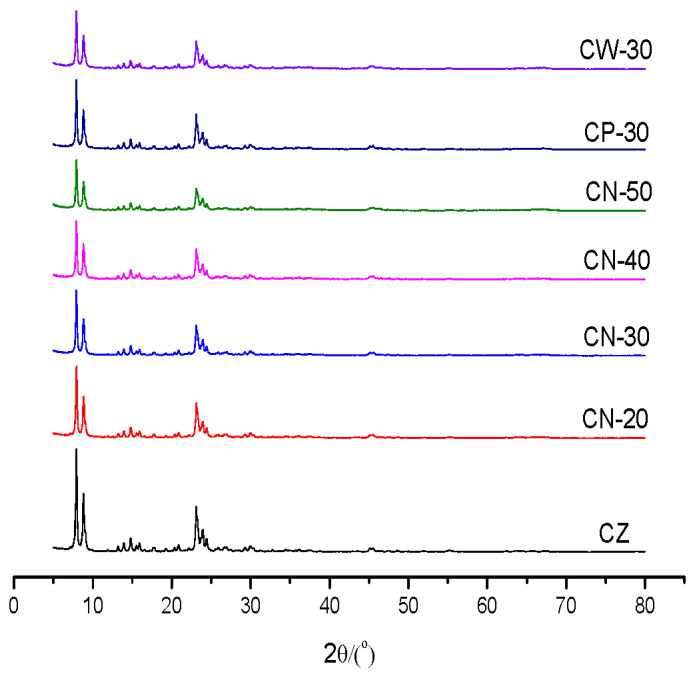
XRD patterns of the catalysts.

The XRD patterns of the catalysts are shown in Figure 1. It can be concluded that all the samples have MFI characteristic structure diffraction peaks. There is no impurity in sample CZ, which indicates that the synthesized crystal is ZSM-5 zeolites. With an increase in binder content the content of HZSM-5 in the unit mass catalyst decreased, and the intensity of the MFI structure characteristic diffraction peak decreased. The diffraction peak intensities of CN-30, CP-30 and CW-30 are slightly different: the diffraction peak intensities of CP-30 are higher than those of CN-30, followed by those of CW-30, which indicates that the molding method has some effect on crystal diffraction peak intensity; dilute nitric acid does not cause obvious damage to the crystal structure of HZSM-5 zeolites.

**Figure 2:**
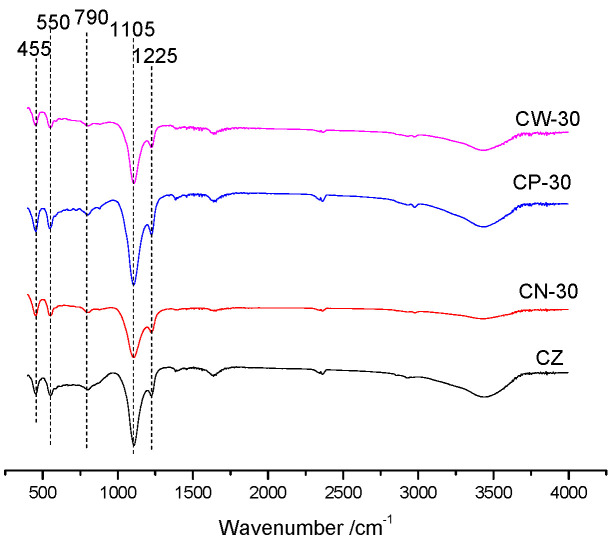
FT-IR spectra of the catalysts’ structure.

The FT-IR spectra of the catalysts are shown in Figure 2. It can be seen that the synthesized zeolite has characteristic peaks at about 455, 550, 790, 1105 and 1225 cm^−1^. The band near 455 cm^−1^ reflects the T-O-T bending vibration, the band near 550 cm^−1^ reflects the bending vibration of five-membered ring in the structural of ZSM-5, the band near 790 cm^−1^ reflects the T-O-T out-of-plane symmetric stretching vibration, the band at 1105 cm^−1^ reflects the T-O-T antisymmetric stretching vibration of tetrahedrons and the band at 1225 cm^−1^ reflects the antisymmetric stretching vibration of inner tetrahedrons. The catalysts appeared to have FT-IR peaks characteristic of ZSM-5 zeolite, which shows that the synthesized samples have an MFI structure.

**Figure 3:**
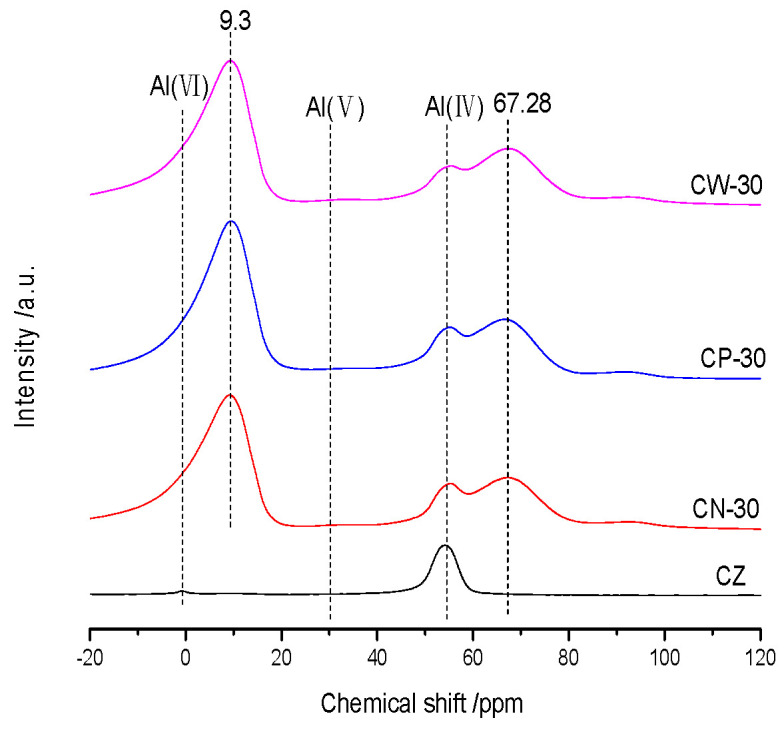
^27^Al NMR spectra of the catalysts.

The ^27^Al NMR spectra of the catalysts are shown in Figure 3. The resonance peak at 54 ppm is designated as tetrahedral coordination framework aluminum (FAL), and the weak peak at 0 ppm is designated as octahedral coordination extra framework aluminum (EFAL) [15]. The peak of catalyst CZ at 54 ppm is sharp and its strength is very high, indicating that most of the aluminum in HZSM-5 raw powder belongs to framework aluminum. The peak of catalyst CZ at 0 ppm is very weak, which may be attributed to the removal of the framework aluminum of HZSM-5 zeolites during the high-temperature calcination step [16,17]. The signal at 54 ppm of CN-30, CP-30 and CW-30 is still clear, which corresponded to tetrahedral coordination FAL, indicating that the crystal structure of HZSM-5 is not destroyed in the molding process. There are two other obvious resonance signals near 9.3 ppm and 67.28 ppm existing in CN-30, CP-30 and CW-30, which are attributed to tetrahedral and octahedral alumina species in γ-Al_2_O_3_, which was formed by the calcination of a pseudoboehmite binder [18,19].

**Figure 4:**
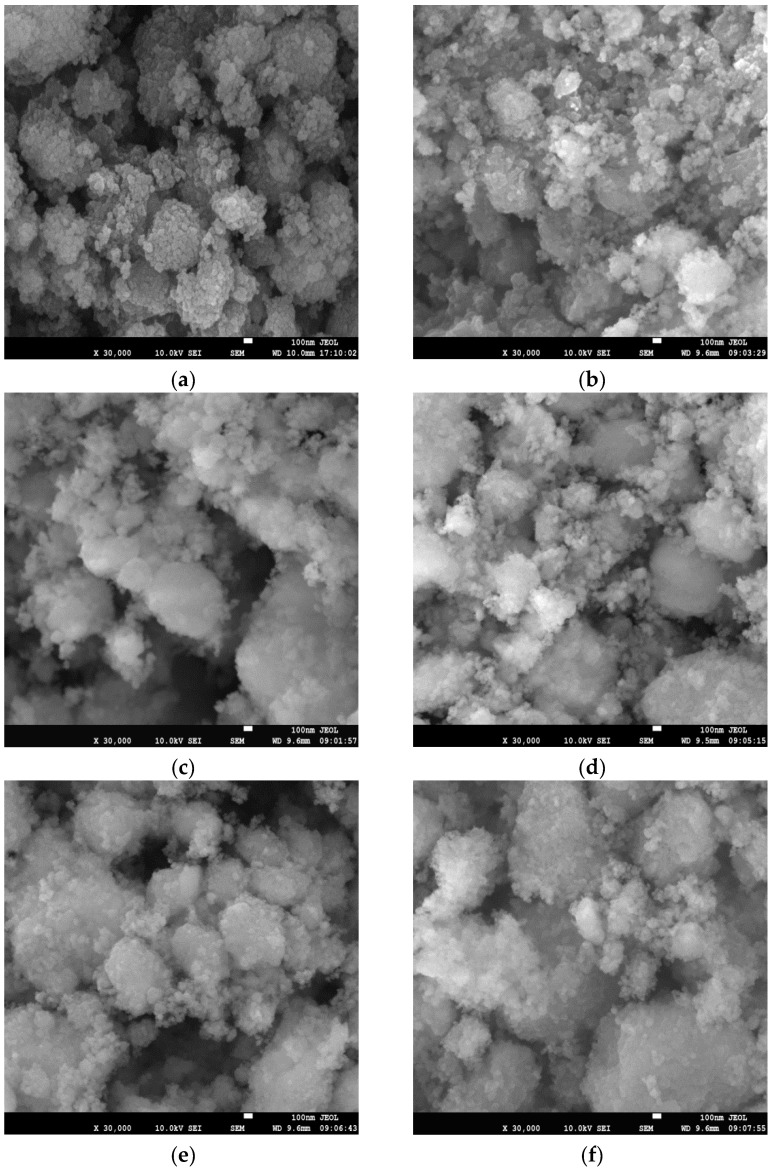

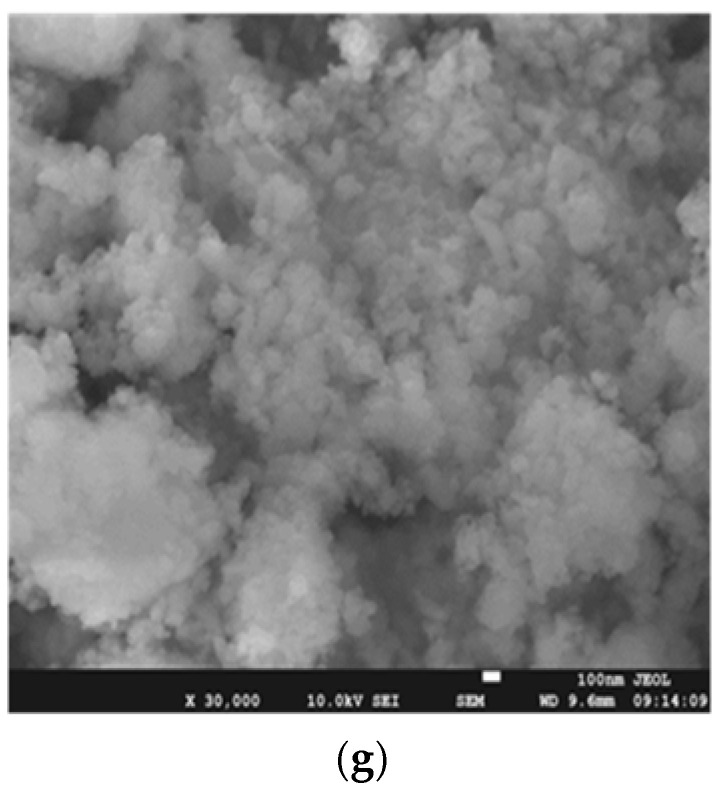
SEM photographs of the catalysts: (**a**) CZ; (**b**) CN-20; (**c**) CN-30; (**d**) CN-40; (**e**) CN-50; (**f**) CP-30; and (**g**) CW-30.

SEM photographs of the catalysts are shown in Figure 4. The crystal size of HZSM-5 is less than 100 nm. The morphology of the catalyst is affected by the molding method: the binder agglomerates with HZSM-5 crystals to form large particles. When the content of the binder is less, pseudoboehmite powder is evenly dispersed in the middle of HZSM-5 zeolite crystals. The particle of CN-20 is nanosized, and its crystal distribution is uniform. With an increase in binder content the agglomerates of the binders form more large particles. There are more large particles in CP-30, followed by CN-30; the particle distribution of CW-30 is relatively uniform.

**Figure 5:**
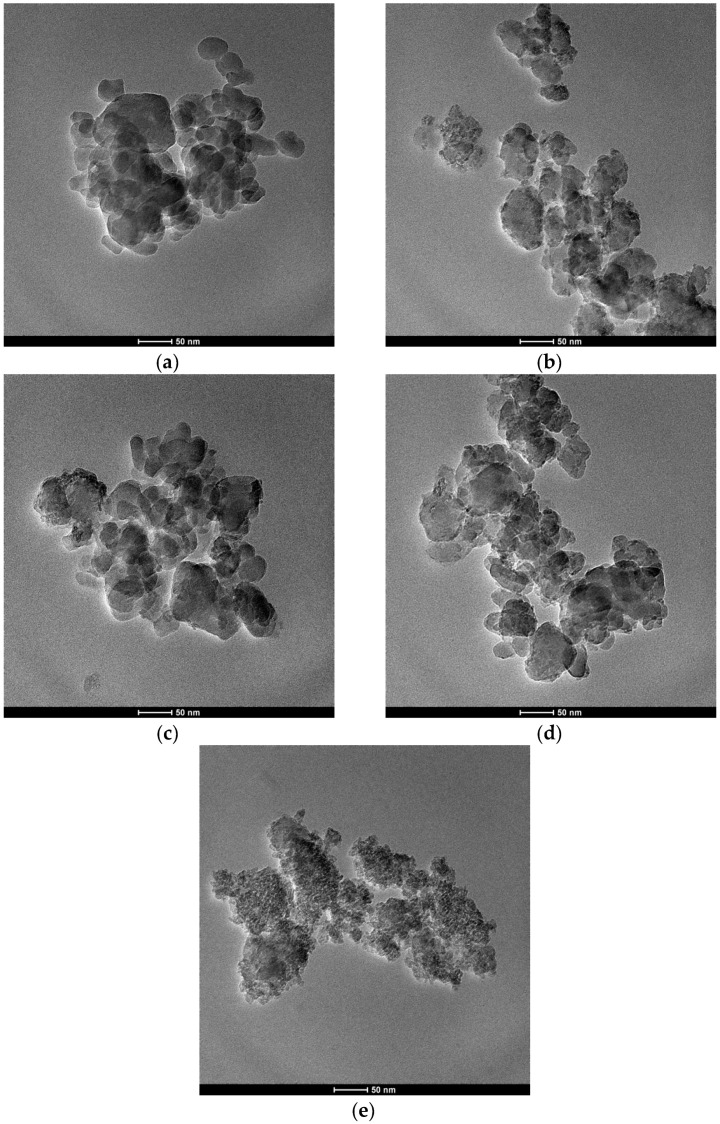
TEM images of the catalysts: (**a**) CZ; (**b**) CN-30; (**c**) CP-30; (**d**) CW-30; and (**e**) binder.

TEM photographs of the catalysts are shown in Figure 5. Most HZSM-5 crystals are less than 50 nm; the binder is amorphous. The binder distribution of the catalysts prepared by the wet extrusion method with dilute nitric acid is more uniform.

### 3.2. Catalyst Acidity

**Figure 6:**
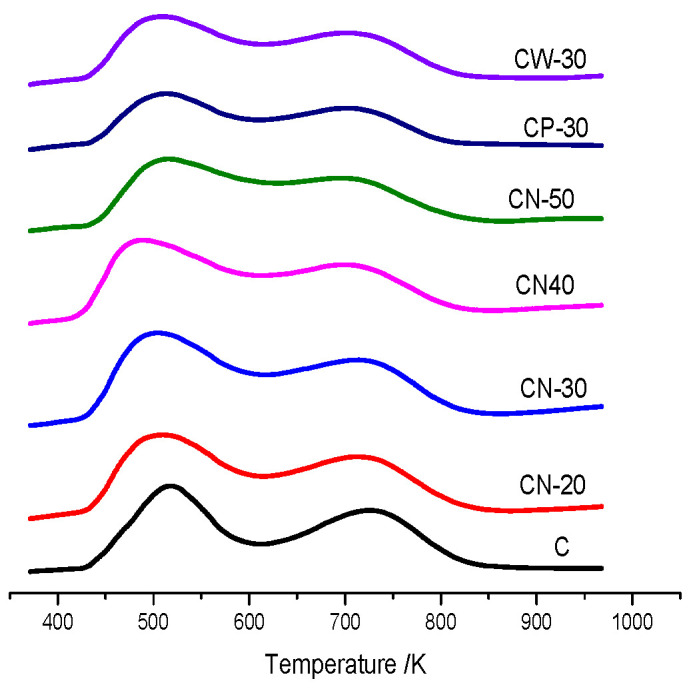
NH_3_-TPD spectra of the catalysts.

The NH_3_-TPD spectra of the catalysts are shown in Figure 6. The peak temperature of weak acid is about 518 K, and that of strong acid is about 723 K on HZSM-5 raw powder CZ. The peak temperature of weak and strong acids decreases by adding different binder contents. The addition of the binder reduces the content of HZSM-5 raw powder in the unit mass catalyst, adjust acid strength and regulates acidity distribution, which is due to the dilution of HZSM-5 zeolite components. With an increase in binder content the weak acid peak temperature of CN-20, CN-30 and CN-40 shifts to a lower temperature in turn; the weak acid peak temperature of CN-50 is higher than that of CN-30 and CN-40, and close to that of CN-20. The weak acid amount of CN series catalysts first increases and then decreases with an increase in binder content. The weak acid peak area of CN-30 is the largest, which indicates that the weak acid amount of CN-30 is the largest. The strong acid peaks of CN series catalysts shift to low temperatures and become wider, which indicates that strong acid strength of CN series catalysts decreases with binder addition. The peak areas of strong acid decrease with the increase of binder content, indicating that the amount of strong acid decrease, while the amount of weak acid and medium strength acid increase. The weak acid peak temperature of CN-30 and CP-30 is close, higher than that of CW-30, which indicates that the weak acid strength of water wet extrusion catalyst is low. The results show that the addition of nitric acid in the extrusion process can form more weak and medium-strength acid centers. The interaction between the binder and HZSM-5 zeolite powder is weak, so the acid strength of CW-30 and CP-30 retains more, while their acid density decreases significantly. Strong acid peaks of CN-30, CP-30 and CW-30 stay at a similar temperature and have similar peak types, while the strong peak area of CN-30 is significantly larger than that of CW-30 and CP-30, indicating that molding methods have different effects on the amount and density of strong acid. Adding dilute nitric acid can promote the interaction between pseudoboehmite and HZSM-5 zeolites, redistribute acid properties and increase the medium-strength acid amount of the catalyst efficiently [20,21].

**Figure 7:**
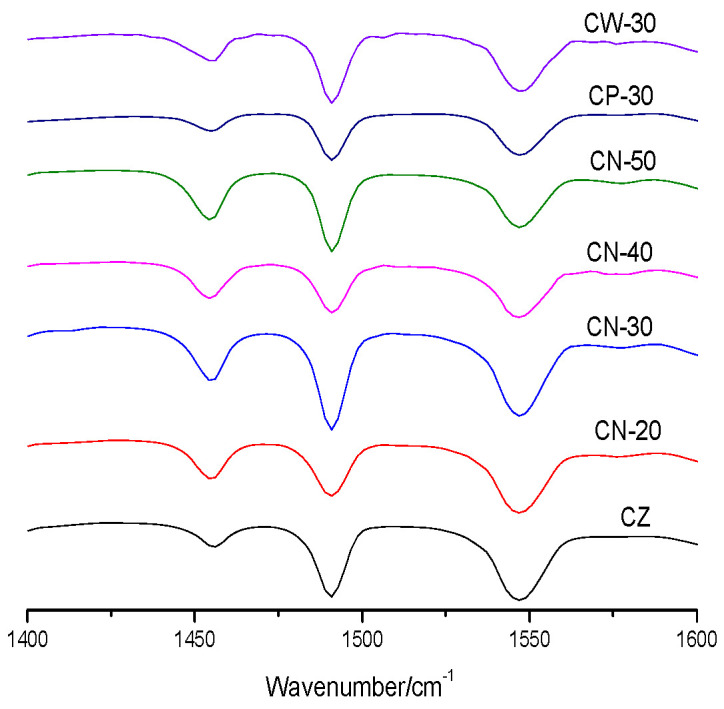
Py-FTIR spectra of the catalysts.

The Py-FTIR spectra of the catalysts are shown in Figure 7. There are three kinds of C-C bending vibration peaks formed by pyridine adsorption in the catalysts, of which the peak near 1545 cm^−1^ represents B acid and the peak near 1454 cm^−1^ represents L acid. The characteristic peaks of the two kinds of acids appeared on all the catalysts, and the peak area changed differently. The B acid peak area of CZ is larger than that of L acid, which indicates that the density of the B acid center on raw HZSM-5 powder is higher than that of L acid. The L acid peak area of CN series catalysts is significantly higher than that of CW-30 and CP-30, which indicates that acid types are redistributed by wet extrusion molding with dilute nitric acid. The acid peak area and intensity of CN-30 were significantly higher than those of other catalysts, which prove once again that the addition of dilute nitric acid may cause a chemical reaction between HZSM-5 zeolites and the pseudoboehmite binder, resulting in the formation of new acid centers, the changing of acid amounts and the redistribution of acid strength [22].

By using ICP characterization the aluminum content of CZ is measured as 1.18%, and the silicon content is 45.64%; therefore, the Si/Al ratio of CZ is 37.30. In Table 1 the NH_3_-TPD and Py-FTIR quantitative results of the catalysts are shown. The relationship between the total acid amount and binder content is not obvious after adding the binder, while the molding method has an obvious effect on acid distribution, indicating that different methods cause different interactions between the binder and HZSM-5 raw powder. The quantitative acid results of NH_3_-TPD show that the total acid amount first increases and then decreases gradually with an increase in the binder content. The acid amount of CN-30 is the highest, at 0.73 mmol NH_3_/g. The total acid amount of CN-20 and CN-40 is about 0.65 mmol NH_3_/g; that of CN-50 and CP-30 is similar, 0.56–0.57 mmol NH_3_/g; and CW-30 is the least, at only 0.45 mmol NH_3_/g. The results above show that the acid density of CN-30 is the highest and that of CW-30 is the lowest. The total acid amount of CN-20, CN-30 and CN-40 is higher than that of CZ; however, the acid strength of CN series catalysts decreases combined with NH_3_-TPD spectra. As mentioned above, this may be due to the chemical interaction between the binder and HZSM-5 zeolite powder by adding dilute nitric acid to the wet extrusion process, resulting in the formation of new acid centers [23]. No matter what molding method is used, the acid strength distribution is greatly modified by adding a binder of pseudoboehmite.

The results of the B/L ratio calculated by Py-FTIR characterization show that CZ has the highest B/L ratio, whose B acid is 4.63 times higher than L acid. The ratio of B acid to L acid gradually decreases with an increase in the binder content; the B/L ratio of CN-50 is the lowest, which decreases to 1.42. The B/L ratios of CP-30 and CW-30 are close, both about 3.6, lower than that of CZ and higher than that of CN-30. The molding method has great influence on the acid type distribution, which can effectively reduce the B acid density and the B acid strength. The addition of dilute nitric acid in the molding process is more conducive to reducing the B acid ratio. Maybe there is a solid-state reaction between the binder and HZSM-5 zeolite powder under acidic extrusion conditions; it is more conducive to the close combination of the two kinds of powder, so the modification effect of the B acid center on the surface of raw powder is stronger, or dilute nitric acid plays a role in removing framework aluminum to some extent [24]. The catalysts prepared by mechanical mixing and the wet extrusion method with water have weak interaction between HZSM-5 zeolites and the binder, so a strong B acid center of CP-30 and CW-30 is retained more.

### 3.3. Catalysts Pore Structure

**Figure 8:**
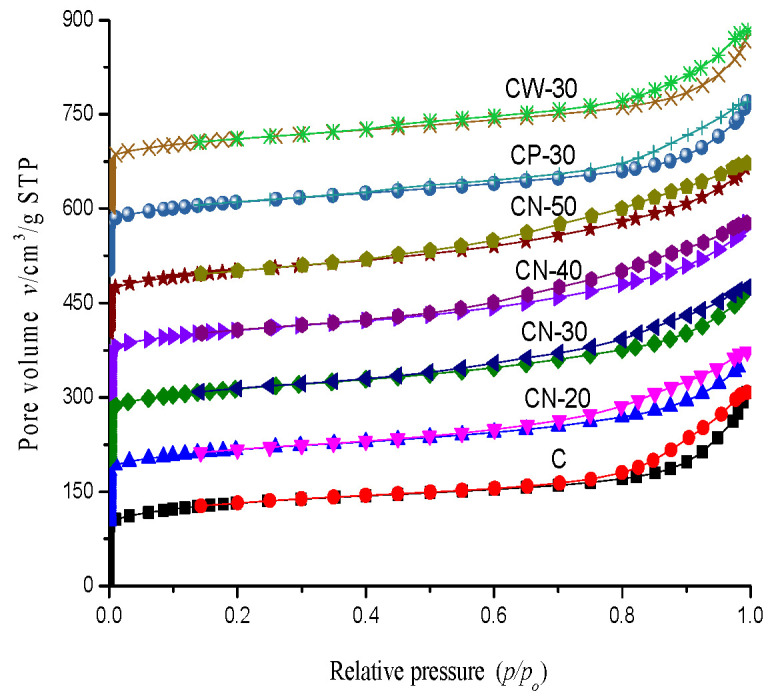
The low-temperature N_2_ adsorption and desorption isotherms of the catalysts.

The low-temperature N_2_ adsorption and desorption isotherms of the catalysts are shown in Figure 8. The adsorption isotherms and desorption isotherms of all the catalysts do not coincide, and the hysteresis loops appear, indicating that there are a certain number of mesopores in the catalysts. The hysteresis loop of CZ is slightly larger, mainly in the region of higher relative pressure. The hysteresis ring became slender and shifted to low pressure after adding the binder. The results show that pore distribution changes with preparation methods, after which secondary intercrystal pores of different shapes are formed.

The low-temperature N_2_ adsorption and desorption results of the catalysts are shown in Table 2. The raw powder HZSM-5 has a high BET surface area, reaching 481.65 m^2^/g. With an increase in binder content, the BET surface area and micropore surface area of the catalysts gradually decreases. The order of external surface area is CZ > CN-50 > CN-30 > CN-4 ≈ CP-30 > CW-30 > CN-20, and the changeable rule of the external surface area with the binder content and molding method is not obvious. The external surface area of CN-20 is the minimum, and that of CN-50 is the maximum. There is a little difference in the external surface area, which is no more than 20 m^2^/g. The total pore volume of CZ is the largest, reaching 0.4763 cm^3^/g. The total pore volume decreases with the addition of the binder, the micropore volume decreases with an increase in binder content and the mesopore pore volume slightly increases with an increase in binder content. The pore size of γ-Al_2_O_3_ obtained by calcined pseudoboehmite is larger than that of HZSM-5 zeolites, and some pseudo boehmite binders may block the micropores; therefore, the micropore volume of the catalyst decreases and the mesopore volume increases gradually with an increase in binder content [25]. The BET surface area, micropore surface area and micropore surface area of the CN-30, CP-30 and CW-30 catalysts prepared by three different molding methods have little difference, with the maximum difference being 10.39 m^2^/g. The decreasing degree of micropore volume and mesopore volume of CP-30 is higher than that of CN-30 and CW-30, which indicates that the mechanical mixing method has a more obvious influence on pore structure than the other two methods. The mechanical mixing method is not conducive to the formation of mesopores and is easier to block micropores with. Because the crystal of HZSM-5 zeolites used in this experiment is nanometer size, the largest intercrystal pore has been formed by nano-HZSM-5 crystals, which leads to a lesser degree of influence of the pseudoboehmite binder on the total pore volume. The surface of the HZSM-5 crystal was covered by the binder, resulting in a decrease in micropore volume. A large amount of mesopores can be formed by nano-HZSM-5 crystals; the mesopore volume is mainly contributed by intercrystal mesopores of HZSM-5 zeolites. Although the mesopore volume gradually increases with an increase in binder content, it is still lower than the intercrystal mesopores formed by HZSM-5 raw powders.

**Figure 9:**
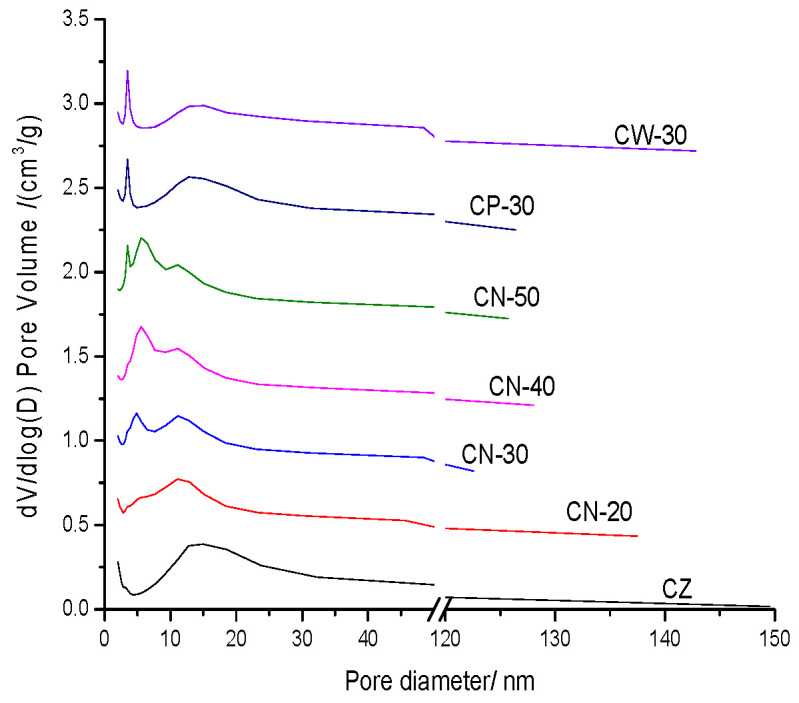
Pore distribution of the catalysts.

The pore distribution of the catalysts is shown in Figure 9. It can be concluded that the binder content and molding method have an important influence on the pore size distribution of catalysts [26]. In addition to the micropore of HZSM-5 itself there is a concentrated mesopore distribution formed at about 15 nm for CZ, which is due to the formation of large intercrystal mesopores. The concentrated mesopore distribution of CN-20 shifted to a low pore diameter, indicating that the addition of the binder changed the interaction of HZSM-5 crystals. There are two kinds of concentrated pore distribution at about 4.79 nm and 11.28 nm for CN-30 in addition to 5.57 nm and 11.28 nm for CN-40 and CN-50. Besides, there is a concentrated pore distribution at about 3.45 nm for CN-50, which may be formed by the binders due to the high binder content. With an increase in binder content a hierarchical pore distribution was formed. Different from CN series catalysts, CP-30 and CW-30 have similar pore distribution, with two kinds of concentrated pore distribution at about 3.45 nm and 13.84 nm. The number of pores at 3.45 nm for CP-30 and CW-30 is higher than CN-30, which is similar to CN-50; this may be due to the weak interaction of binders with HZSM-5 crystals and the strong interaction of binders. A pore distribution comparison showed that dilute nitric acid plays an important role in the pore formation of the molding process, increasing hierarchical pore distribution, which is more conducive for improving diffusion performance [27].

### 3.4. Catalyst Surface Characterization

**Figure 10:**
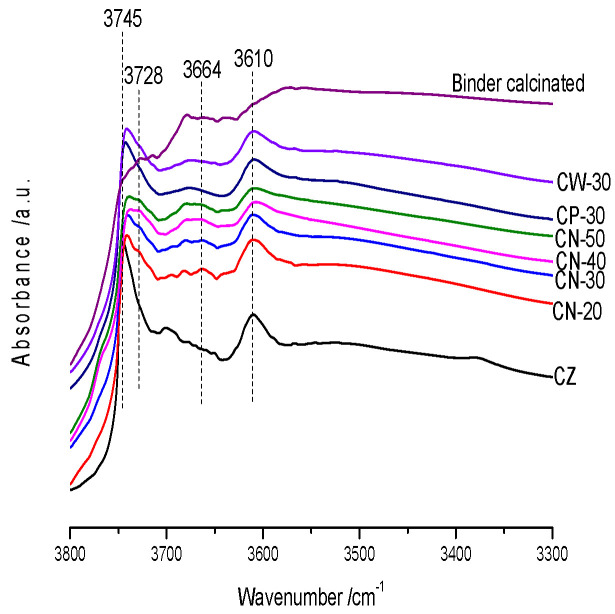
FT-IR spectra of the catalysts’ surface O-H stretching vibrations.

The FT-IR spectra of the catalysts’ surface O-H stretching vibrations are shown in Figure 10. The band at 3745 cm^−1^ is related to isolated external silanols [Si-OH] and the shoulder towards lower frequencies (3728 cm^−1^) has been assigned to weakly interacting internal silanols, while the band at 3610 cm^−1^ is assigned to B acid sites associated with framework aluminum [Si-(OH)-Al], typically located at the external surface. The band at 3500 cm^−1^ is related to delocalized hydrogen-bonded groups of lattice defects. The additional weak band located at 3664 cm^−1^ is observable at catalysts with the binder, which is assigned to extraframework aluminium (EFAl) species [15,28]. The characteristic peaks of external silanols [Si-OH] (3745 cm^−1^) and skeleton aluminum [Si-(OH)-Al] (3610 cm^−1^) on the catalysts are obvious. With the addition of the binder the peak intensity of external silanols [Si-OH] (3745 cm^−1^) decreases and widens. With an increase in binder content the external silanols [Si-OH] peak widens more severely and the internal silanols shoulder peak (3728 cm^−1^) appears, which indicates that the silanols become dispersed and more abundant. The framework Al [Si-(OH)-Al] (3610 cm^−1^) peak remained on the formed catalyst. When the binder content reached 40% the peak of 3610 cm^−1^ was significantly widened. The [Si-(OH)-Al] (3610 cm^−1^) peaks of CN-30, CP-30 and CW-30 have little difference, and the peak distribution of the external silanols [Si-OH] (3745 cm^−1^) of CP-30 is more concentrated, followed by CW-30. The peak distribution of the external silanols [Si-OH] (3745 cm^−1^) of CN-30 is wider than that of CP-30 and CW-30, indicating that the addition of dilute nitric acid is conducive to enhancing the interaction between the binder and HZSM-5 zeolites.

**Figure 11:**
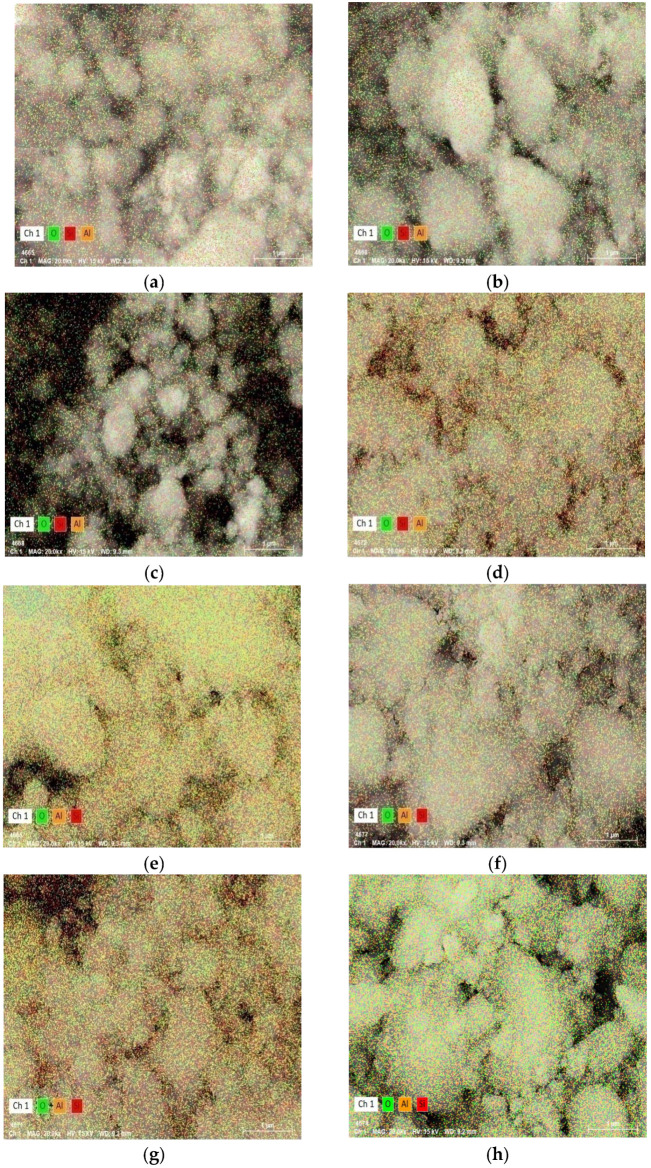
SEM-EDS elemental mapping of the catalysts: (**a**) CZ; (**b**) CN-20; (**c**) CN-30; (**d**) CN-40; (**e**) CN-50; (**f**) CP-30; (**g**) CW-30; and (**h**) binder.

The element distribution results of the catalysts from SEM-EDS are shown in Figure 11 and Table 3. With an increase in binder content the aluminum content increases, and the silicon content first increases and then decreases. The aluminum content of CN-30 and CW-30 is close, about 9.4%. The aluminum content of CP-30 is less, and the silicon content of CP-30 and CW-30 is higher than CN-20. They show that the binder and HZSM-5 distribution of CN-30 is relatively uniform.

### 3.5. Catalyst Evaluation

**Figure 12:**
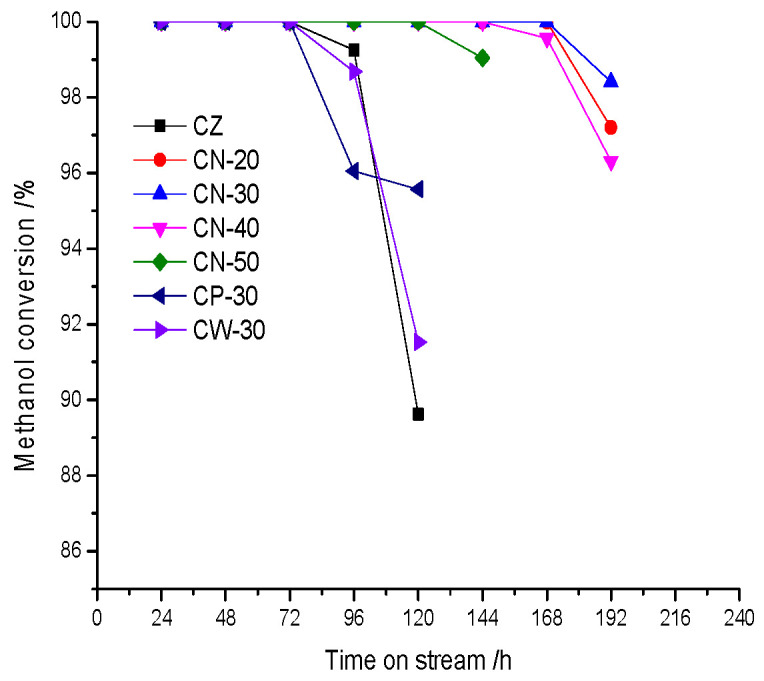
The change in methanol conversion with time on stream.

The catalysts are evaluated under the conditions of atmospheric pressure, 653 K and a methanol WHSV of 4 h^−1^. The change in methanol conversion with time on stream is shown in Figure 12. The presence of methanol in the aqueous phase is the sign of catalysts’ deactivation; it can be concluded that the order of activity stability is CN-20 = CN-30 > CN-40 > CN-50 > CP-30 = CW-30 = CZ. The lifetime of CN-20 and CN-30 is 168 h, followed by CN-40 with 144 h. The lifetime of CZ, CP-30 and CW-30 is only 72 h. When methanol appeared in the aqueous phase methanol conversion decreased at different rates. The methanol conversion of CN-20, CN-30, CN-40 and CN-50 decreased slowly, indicating those whose deactivation rate was slower than other catalysts and that the deactivation rate of CN-30 was the slowest, indicating that its activity stability was the highest. The methanol conversion of CZ, CP-30 and CW-30 decreased rapidly, indicating those whose deactivation rate is very quick. Overall, the molding method of CN-30 is the best and the binder content of 30% is the most suitable; the lifetime is increased by 96 h under the evaluation conditions mentioned above.

**Figure 13:**
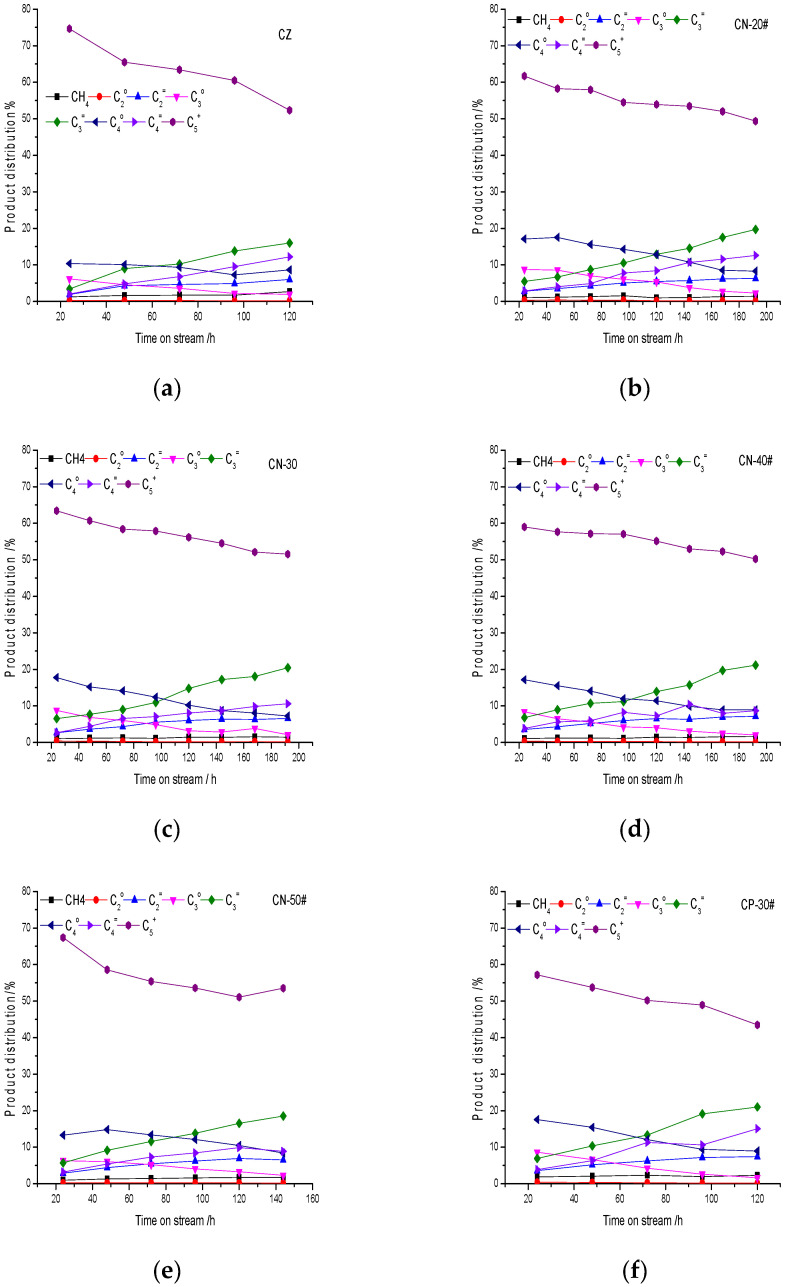

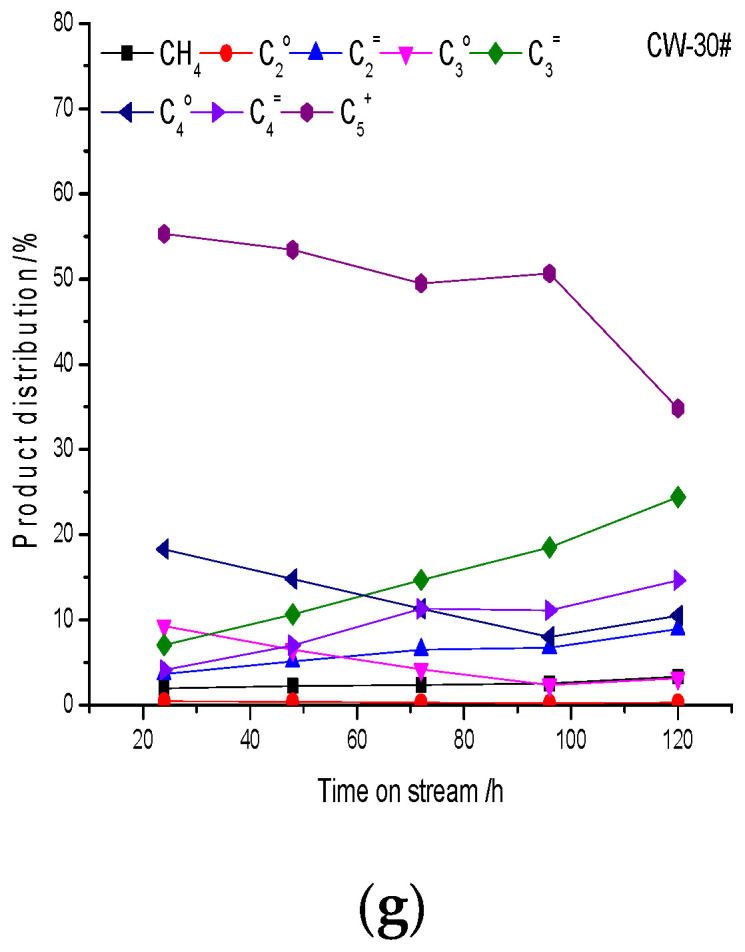
Hydrocarbon product distribution of MTH over the catalysts with time on stream (**a**) CZ; (**b**) CN-20; (**c**) CN-30; (**d**) CN-40; (**e**) CN-50; (**f**) CP-30; and (**g**) CW-30.

The hydrocarbon product distribution of MTH over the catalysts with time on stream is shown in Figure 13. In the range of the active period (methanol conversion is 100%) the selectivity of light olefin (C_2_^=^, C_3_^=^ and C_4_^=^ alkenes) increases, low-carbon alkanes (C_2_°, C_3_° and C_4_° alkanes), except methane, decrease and the selectivity of C_5_^+^ decreases gradually with time on stream.

**Figure 14:**
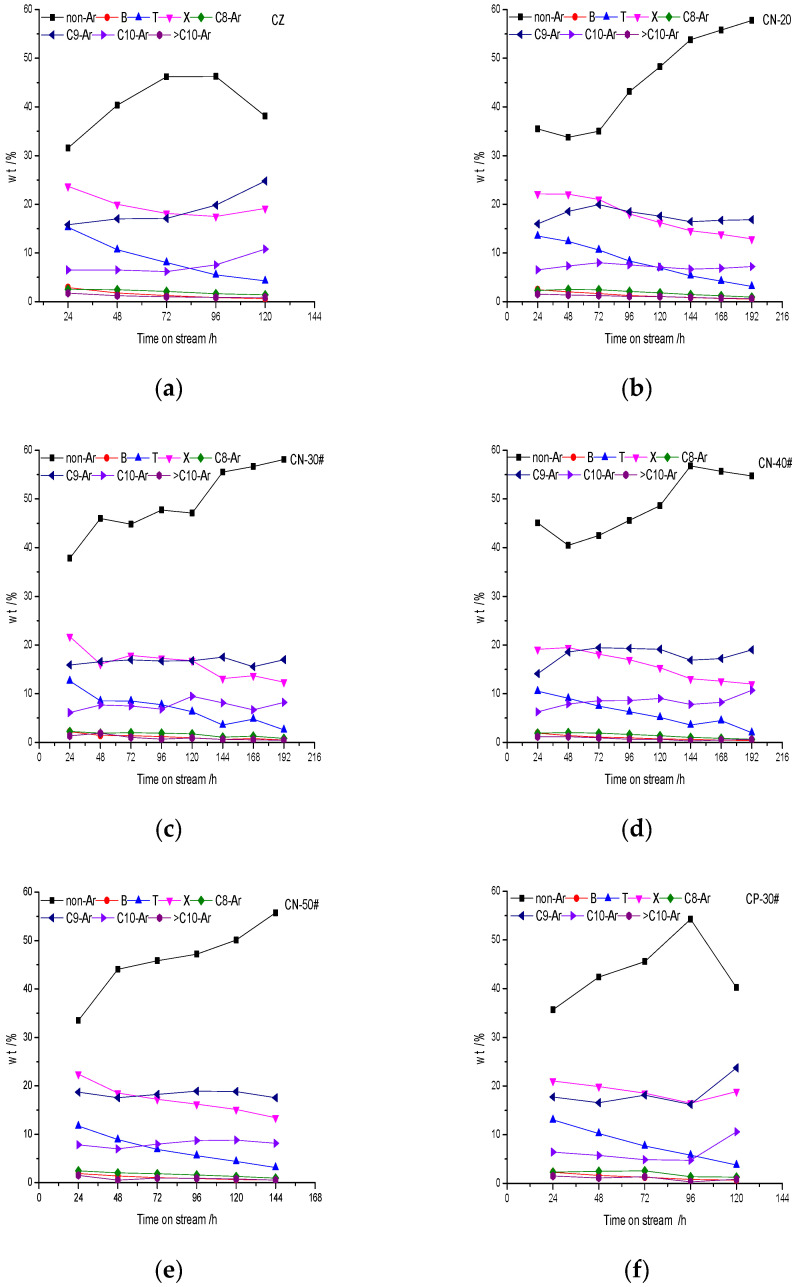

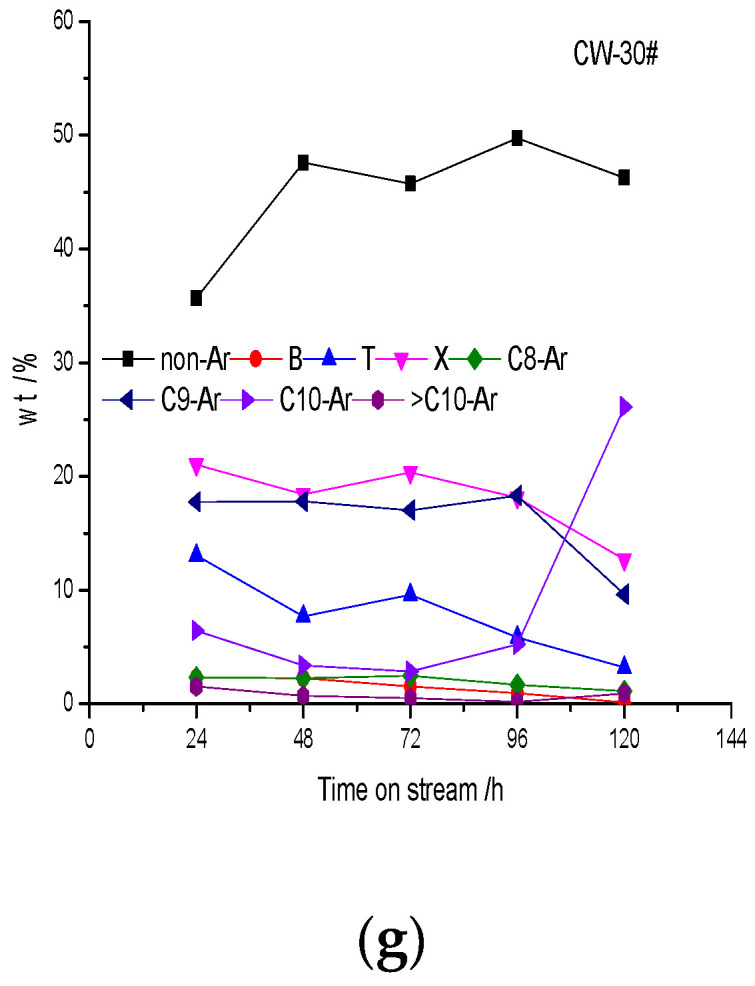
The change in oil composition with time on stream: (**a**) CZ; (**b**) CN-20; (**c**) CN-30; (**d**) CN-40; (**e**) CN-50; (**f**) CP-30; and (**g**) CW-30.

The change in oil composition with time on stream is shown in Figure 14. It showed that the contents of nonaromatics and aromatics in the oil phase change with time on stream constantly. Nonaromatic hydrocarbons are mainly C5–C7 alkanes and olefins, and aromatics are mainly C7–C10 aromatics. In the range of the active period the content of nonaromatics increases gradually, while the content of aromatics decreases gradually.

**Figure 15:**
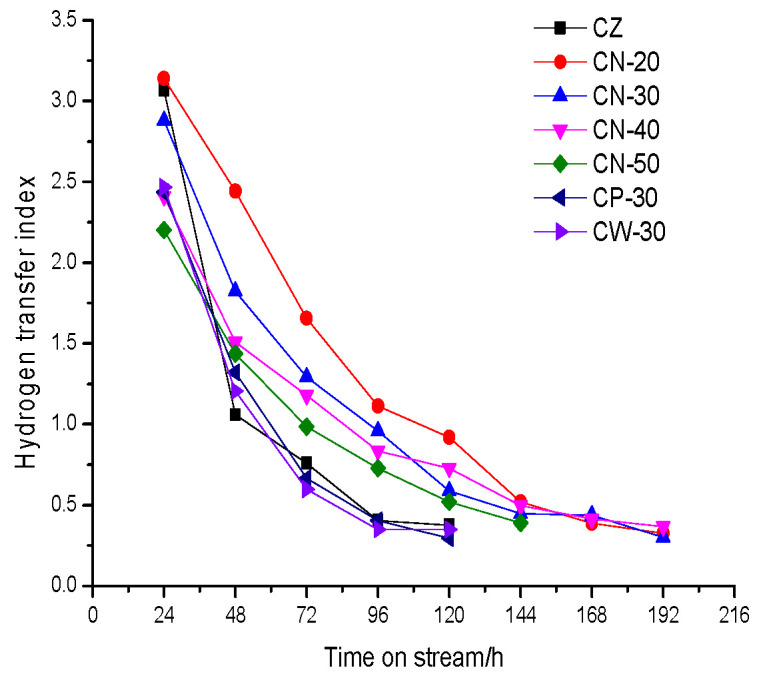
The change in the hydrogen transfer index with time on stream over the catalysts.

The change in the hydrogen transfer index (HTI) with time on stream over the catalysts is shown in Figure 15. It can be seen that the HTI of all the catalysts decreases continuously with time on stream and that the downward trend gradually slows down. At the beginning of the reaction the HTI of the catalyst with less binder content is higher, and the HTI of CW-30 and CP-30 are close, lower than that of CN-30. The HTI of CZ decreased to 3.07 at 24 h and 1.06 at 48 h. Its reduction speed is the fastest, followed by CW-30; the third is CP-30; the fourth is CN-30; and CN-20 and CN-50 decreased slowly.

The results of the coke, C and H content of the coke-deposited catalysts are shown in Table 4. The coke weight loss of CZ is the most, at 27.41%, indicating that its coke deposition amount is the largest; additionally, its C/H ratio of coke deposition is the highest, reaching 1.75, while the lowest C/H ratio was of CN-50, at 1.36. The coke deposition amount of CN series catalysts first increases and then decreases from CN-20 to CN-50; CN-30 has the highest coke deposition amount, which is due to it having the longest lifetime. The C/H ratios of CN-20, CN-30 and CN-40 are the same, 1.53, while the C/H ratio of CN-50 decreases significantly. The C/H ratio order of the catalysts prepared by the three methods is CP-30 > CW-30 > CN-30.

## 4. Discussion

CN-30 has the best catalytic performance. It suggested that adding a binder and wet extrusion molding with dilute nitric acid can effectively reduce the deactivation rate and improve activity stability. As we all know, coke deposition is the main reason of one-way deactivation in the MTH reaction and the catalyst activity can be restored by burning coke deposition. Strong B acid sites are conducive to the reactions of polymerization, cracking, cyclization and hydrogen transfer, which eventually generate macromolecular polycyclic aromatics, and they are the active centers of deep coke deposition. The addition of the binder not only increases the mechanical strength of the catalyst but also disperses and covers strong B acid sites [29]. A strong acid amount and strong acid strength decreased significantly with the addition of the binder, and strong acid strength decreased gradually with an increase in binder content. On the one hand, although the acid amount of CN-30 increases and its acid density is the highest, the acid strength of CN-30 decreases significantly and its proportion of B acid is moderate. On the other hand, there are two kinds of concentrated pore distribution in CN-30, forming a hierarchical pore structure that greatly improves diffusion performance. CN-30 therefore has the best activity stability due to the combination effect of acidity and pore distribution. The B acid ratio of CP-30 and CW-30 obtained by the mechanical mixing method and water extrusion method is higher, after which a deep coke deposition reaction is more likely to occur; therefore, the deactivation is faster.

NH_3_-TPD and Py-IR characterizations show that CZ has strong acidity, high acid density and the highest proportion of B acid, which makes it easier to deactivate via carbon deposition. However, the activity stability of CZ is the same as that of CP-30 and CW-30, which is 72 h, methanol conversion of CZ decreases more rapidly at 96 h than that of CP-30 and CW-30, and begins to decrease sharply at 120 h. This indicates that pore structure also has some certain influence on activity stability. CZ molded by the pressing of HZSM-5 zeolites into tablet has formed a large number of mesopores and macropores via nano-HZSM-5 crystals, which improves diffusion performance and is conducive to the diffusion of precursors of macromolecular coke deposition; therefore, the deactivation rate of CZ is reduced to a certain extent. The proportion of B acid decreases with an increase in binder content, indicating that the strength and density of B acid decreased significantly, while the proportion of B acid of CP-30 and CW-30 is higher than that of CN series catalysts, which aggravated the deep coke reaction. Pore structure data show that the micropore volume and micropore surface area of CP-30 and CW-30 decreased, which indicates that the binder covered the surface and pore entrance of HZSM-5 zeolites in addition to blocking the micropore. The pore distribution also confirms the above statement. Consequently, the catalysts prepared by the mechanical mixing method and water wet extrusion method are detrimental to the diffusion of macromolecular products. The activity stability of CP-30 and CW-30 is not improved, and the activity stability is close to that of CZ with the effect of the acid distribution and pore structure.

According to the “hydrocarbon pool” reaction mechanism, methanol first forms “hydrocarbon pool” active species, after which methanol and “hydrocarbon pool” active species continue to react to generate various hydrocarbon products. The “hydrocarbon pool” species on HZSM-5 are mainly long-chain olefins, polymethylbenzene and cyclopentene carbon cations [30,31]. These species are also important deactivate intermediates, and they can also be called coke deposition precursors. With the progress of the reaction the coke deposition on the catalyst gradually increases, covering the surface of the acid center, reducing the acid strength and acid density; therefore, the capacity of polymerization, cracking, cyclization and hydrogen transfer gradually decreases, the olefin content gradually increases, the alkane content gradually decreases and C_5_^+^ hydrocarbon products decrease gradually. In the same way, the catalysts with strong acidity, high proportions of B acid centers and large acid amounts have strong abilities of polymerization, cracking, cyclization and hydrogen transfer, so olefins are lower and alkanes are higher. The acid center is the active center of the reaction, especially the strong B acid center, which is the key factor of the reaction performance. If acidity is strong and the acid density is high the combination of “hydrocarbon pool” species and acid sites is strong, and products will not be easy to desorb from catalysts, resulting in deep coke deposition. If the acidity is moderate methanol can continuously react with “hydrocarbon pool” species, and the catalyst will have a long lifetime. Gas-phase hydrocarbon products of the catalysts with the addition of the binder increased, while high-carbon-number C_5_^+^ hydrocarbon products increase gradually, which is mainly due to the decrease in strong acid strength and acid density.

The oil composition change is also mainly due to the effect of coke deposition on the acidity during the reaction. As mentioned above, with an increase in coke deposition the acid center is covered by coke deposition, acid strength decreases, acid density decreases and the ability of polymerization and cyclization decrease. Therefore, the content of nonaromatic hydrocarbons with a low carbon number increases and the content of aromatics decreases. Polymethylbenzenes (toluene, xylene, trimethylbenzene and tetramethylbenzene) are the main aromatics. The content of aromatics with carbon number larger than C10 is less; the trend is not obvious, which is due to the shape selectivity of HZSM-5 crystal micropores. Benzene, toluene and C8 aromatics are mainly obtained from cracking reactions; the cracking ability is stronger at the initial stage of the reaction, so the content of benzene, toluene and C8 aromatics is higher at first and then decreases gradually, which is due to the influence of coke deposition on acidity. The change in C9 aromatics is not obvious, and the main C9 aromatics component is the thermodynamic equilibrium product meta-trimethylbenzene. C10 aromatics are mainly tetramethylbenzenes, in which the content of durene is the largest, and its content increases with time on stream. As the reaction proceeds the cracking activity is insufficient, so durene increases. The results suggest that the relationship of aromatics distribution and pore distribution is not obvious, which is mainly related to acidity. The acid distribution affects product distribution directly. A catalyst with strong acidity and a high density of acid centers has strong cracking ability, and the content of low-carbon-number aromatics is high. On the contrary, the content of polymethyl aromatics is high.

The change in HTI is also due to carbon deposits formed in the reaction process, which modifies the acidity, weakens strong acid strength and decreases acid density. Therefore, the HTI decreases continuously with the reaction. The trend in the HTI showed that the coke deposition rate of a catalyst with a high B/L ratio was very fast. The addition of the binder can reduce the acid strength, make the distribution of acid centers more dispersed and reduce the density of acid centers, which can delay coke deposition; therefore, the HTI of the catalysts with a high binder content decreased slowly. The HTI corresponds to the difference in catalysts’ acidity, which is related to the B/L ratio and acid density directly. The essential reason is that the molding methods and binder content have different effects on acidity, which was mentioned earlier.

Acidity is the main factor that affects the properties of coke deposition; aromatics are prone to deep coking reactions over catalysts with a high acid density and strong acidity. Therefore, the amount of coke deposition of CZ is the largest and the C/H ratio of coke deposition is the highest. The interaction between the binder and strong acid sites prepared by the mechanical mixing method is weak, its strong acid sites are retained more and the B acid ratio is higher; therefore, its C/H ratio is higher, followed by CW-30. Adding dilute acid to the molding process of CN-30, which enhances the interaction between the binder and HZSM-5 zeolites, covers strong acid centers, reduces acid strength, and inhibits the deep coking reaction effectively. The trend in the TG and C/H ratio of coke deposition corresponds to acidity changing, which verifies the influence of acidity difference on the reaction performance.

## 5. Conclusions

Molding by adding a binder of pseudoboehmite changes the acid distribution of the catalysts, the amount of medium strength acid increases, the amount of strong acid decreases, the strength of strong acid decreases and the B/L ratio decreases. The medium-strength acid amount of the catalyst molded by wet extrusion with dilute nitric acid is increased and the B acid proportion is moderate, while the mechanical mixing and water wet extrusion methods retained more B acid sites. With the increase in binder content the acid amount first increases and then decreases, and the B/L ratio decreases gradually.

For nano-HZSM-5 zeolite crystals a large number of intercrystal mesopores can be formed by nanocrystals. The addition of the binder can form an obvious hierarchical pore distribution, but the total pore volume decreases. The mechanical mixing method and water wet extrusion molding method cause micropore blockage; the molding method of wet extrusion with dilute nitric acid is conducive to the formation of a more obvious hierarchical pore distribution. Hierarchical pore distribution is beneficial to product diffusion, reducing the deactivation rate of carbon deposition and improving the activity stability of the catalyst.

The addition of dilute nitric acid in the molding process strengthens the interaction of HZSM-5 zeolites and binders, increases the medium-strength acid amount and forms a more obvious hierarchical pore distribution. However, the hierarchical pore structure of the mechanical mixing method and water wet extrusion molding method is not obvious. The acidity of the 30% binder content catalyst prepared by wet extrusion molding with a dilute nitric acid solution is moderate, especially its strong acid strength and B/L ratio, whose hierarchical pore structure is the most obvious; therefore, the activity stability of CN-30 is the highest.

Acid distribution is the main reason for product distribution of the catalysts, which is related to the ability of the hydrogen transfer reaction, the amount of coke deposition, the rate of coke deposition and the C/H ratio of coke deposition. The catalyst with strong acidity and a large strong acid amount has high hydrogen transfer ability, large coke deposition, a fast coke deposition rate and a high carbon C/H ratio. A strong B acid center is conducive to the formation of high-carbon hydrocarbons and aromatics, while pore distribution has little influence on product distribution.

## Figures and Tables

**Table 1:** The NH_3_-TPD and Py-FTIR quantitative results of the catalysts.

Catalysts	Weak Acid ^1^ (mmol NH_3_/g)	Strong Acid ^1^ (mmol NH_3_/g)	Total Acid ^1^ (mmol NH_3_/g)	B/L ^2^
CZ	0.31	0.31	0.62	4.63
CN-20	0.34	0.33	0.67	2.48
CN-30	0.39	0.34	0.73	2.19
CN-40	0.35	0.29	0.64	1.85
CN-50	0.32	0.25	0.57	1.42
CP-30	0.31	0.25	0.56	3.70
CW-30	0.24	0.21	0.45	3.56

^1^: density of acid sites, determined from NH_3_-TPD results; ^2^: determined from 573 K Py-FTIR spectra.

**Table 2:** Low-temperature N_2_ adsorption and desorption results of the catalysts.

Catalysts	S_BET_ /(m^2^·g^−1^)	S_micro_^1^ /(m^2^·g^−1^)	S_ext_^1^ /(m^2^·g^−1^)	V_total_^2^ /(cm^3^·g^−1^)	V_micro_^3^ /(cm^3^·g^−1^)	V_meso_^4^ /(cm^3^·g^−1^)	D_pore_ /(nm)
CZ	481.65	271.72	209.93	0.4763	0.1121	0.3642	3.96
CN-20	423.82	234.74	189.07	0.4221	0.0970	0.3251	3.98
CN-30	409.01	202.15	206.86	0.4248	0.0843	0.3405	4.16
CN-40	384.18	182.40	201.77	0.4283	0.0763	0.3520	4.46
CN-50	360.57	147.72	212.85	0.4205	0.0626	0.3579	4.66
CP-30	398.71	196.96	201.75	0.4173	0.0822	0.3351	4.19
CW-30	401.42	202.83	198.59	0.4373	0.0844	0.3529	4.36

^1^: micropore surface area and external surface area, from *t*-plot; ^2^: single-point adsorption total pore volume of pores; ^3^: micropore volume, from *t*-plot; and ^4^: mesopore volume, from the BJH method.

**Table 3:** Element distribution results of the catalysts from SEM-EDS.

Catalysts	O Content/%	Al Content/%	Si Content/%
CZ	63.3	1.5	35.2
CN-20	64.0	7.6	28.4
CN-30	59.9	9.7	30.4
CN-40	56.7	12.4	30.9
CN-50	55.3	20.0	24.7
CP-30	61.2	5.8	33.0
CW-30	57.6	9.1	33.3
Binder	56.1	43.9	0.0

**Table 4:** The results of the coke, C and H content of the coke-deposited catalysts.

Catalysts	Coke Content ^1^/%	C Content ^2^/%	H Content ^2^/%	C/H^2^ Molar Ratio
CZ	27.41	29.49	1.40	1.75
CN-20	21.46	24.63	1.34	1.53
CN-30	25.24	25.92	1.41	1.53
CN-40	24.87	25.23	1.37	1.53
CN-50	21.20	21.98	1.35	1.36
CP-30	25.37	26.00	1.27	1.71
CW-30	24.42	27.57	1.39	1.65

^1^: coke content, determined from TG results; ^2^: coke component, determined from C and H elements analysis.

## Data Availability

The data presented in this study are available upon request from the corresponding author.
